# Intravascular Lithotripsy Is Associated With Superior Clinical Outcomes Compared to Atherectomy: A Large‐Scale, Propensity‐Matched Analysis

**DOI:** 10.1002/ccd.70464

**Published:** 2026-03-16

**Authors:** Charles D. Miks, Grant K. Ozaki, Chirayu R. Shukla, Vikram Sharma

**Affiliations:** ^1^ Department of Internal Medicine University of Iowa Health Care Iowa USA; ^2^ Division of Cardiovascular Medicine University of Iowa Health Care Iowa USA

**Keywords:** coronary calcification, drug‐eluting stent, percutaneous coronary intervention, plaque modification

## Abstract

**Background:**

Coronary artery disease (CAD) with severe calcification remains a growing challenge in percutaneous coronary intervention (PCI). Atherectomy (ATH) has long been used for plaque modification, whereas intravascular lithotripsy (IVL) has emerged as a promising alternative. Large‐scale comparative data on long‐term outcomes are limited.

**Aims:**

To compare 1‐year clinical outcomes, 30‐day procedural complications, and temporal trends between IVL and ATH use.

**Methods:**

We conducted a retrospective cohort study using the TriNetX Research Network, analyzing de‐identified data from 47 health care organizations. Adults with CAD undergoing single‐vessel PCI with drug‐eluting stent (DES) and either IVL or ATH were included. Outcomes were assessed after propensity matching, and annual utilization trends were evaluated from 2013 to 2024.

**Results:**

We identified 13,499 patients who underwent DES with IVL (*n* = 7026) or ATH (*n* = 6473). After matching, 5768 patients remained in each cohort. At 1 year, IVL was associated with lower risk of all‐cause mortality (RR 0.64; 95% CI 0.58−0.72; *p* < 0.0001), myocardial infarction (RR 0.80; 95% CI 0.66−0.96; *p* = 0.0166), and MACE (RR 0.71; 95% CI 0.62−0.82; *p* < 0.0001). IVL also reduced 30‐day procedural complications (RR 0.77; 95% CI 0.61−0.97; *p* = 0.0271), with less bleeding (RR 0.44; 95% CI 0.31−0.62; *p* < 0.0001) and CA‐AKI (RR 0.71; 95% CI 0.55−0.91; *p* = 0.0068). IVL use increased rapidly and surpassed ATH after 2022.

**Conclusion:**

In this large, real‐world registry, IVL was associated with more favorable 1‐year outcomes and fewer 30‐day complications than ATH. These findings support IVL as a safe and effective alternative for complex coronary lesions, emphasizing the need for validation in randomized trials to assess for causality.

**Abbreviations**
AF/AFL: atrial fibrillation and flutter
ATH: atherectomy
CABG: coronary artery bypass grafting
CAD: coronary artery disease
CA‐AKI: contrast‐associated acute kidney injury
CKD: chronic kidney disease
CM: cardiomyopathy
COPD: chronic obstructive pulmonary disease
CTO: chronic total occlusion
CVA: ischemic stroke
DES: drug‐eluting stent
DM: diabetes mellitus
HFpEF: heart failure with preserved ejection fraction
HFrEF: heart failure with reduced ejection fraction
HLD: hyperlipidemia
HTN: hypertension
ICM: ischemic cardiomyopathy
ISR: in‐stent restenosis
IVL: intravascular lithotripsy
MACE: major adverse cardiovascular events
MI: myocardial infarction
PCI: percutaneous coronary intervention
PVD: peripheral vascular disease
SMD: standardized mean difference
TIA: transient ischemic attack

## 1 Introduction

Coronary artery disease (CAD) remains a significant global public health issue affecting over 250 million individuals worldwide [1]. Severely calcified lesions occur in up to 25%−30% of cases and present an increasing challenge for percutaneous coronary interventions (PCI) [2]. Atherectomy (ATH), which includes rotational, orbital, directional, and laser techniques, is a longstanding method for plaque modification of fibrotic or heavily calcified lesions to facilitate stent delivery, but has been associated with periprocedural risks [3]. Intravascular lithotripsy (IVL) has recently emerged as an attractive alternative to ATH, supported by the pivotal DISRUPT CAD I–IV trials that established its safety and feasibility for treating severely calcified coronary disease [4, 5, 6, 7]. However, large‐scale, head‐to‐head randomized trials comparing the long‐term outcomes of IVL to ATH remain limited and have yet to establish definitive outcome differences between these modalities [8, 9, 10].

Despite a lack of randomized clinical trial data, increasing evidence comparing IVL and ATH outcomes comes from retrospective series and registries. One early meta‐analysis pooled 670 patients across five studies, finding no major differences in clinical outcomes [11]. More recent meta‐analyses include over 2000 patients per cohort and report broadly similar clinical outcomes between IVL and ATH, with IVL showing shorter procedure times, reduced contrast use, and fewer complications [12, 13]. These analyses demonstrate a critical need for updated studies with larger populations.

Given the limited available data, we sought to conduct the largest retrospective cohort study comparing IVL to ATH from a single registry to date. Using the TriNetX Research Network, we analyzed 1‐year clinical outcomes and 30‐day procedural complications among patients undergoing single‐vessel PCI with a drug‐eluting stent (DES) and either IVL or ATH. We additionally evaluated the incidence of IVL and ATH from 2013 to 2024 to characterize the evolving adoption of these modalities.

## 2 Methods

We used the TriNetX global research network, a federated, deidentified patient data registry of over 132 million patients from 108 participating health care organizations across six countries, to perform our analysis. All patients over 18 years old with prior CAD (I25.1) undergoing initial single‐vessel DES (027037(Z,6), 027036(Z,6), 027035(Z,6), 027034(Z,6), 92928) were included, and two cohorts were defined by those undergoing either IVL (C1761, 92972) or ATH (92924, 92933) on the same day as stenting (Table 1). Patients with any IVL codes were excluded from the ATH cohort, and vice versa. Patients who underwent both IVL and ATH on the same day were excluded to prevent any overlap between the groups. Patients with prior revascularization with DES or the presence of in‐stent restenosis (ISR) were excluded from both cohorts. Index events were defined by the date of DES implantation with IVL or ATH. Baseline characteristics were calculated from anytime until 1 day before the index event for both cohorts, and 31 high‐risk features and demographic variables were used for propensity matching.

**Table 1:** ICD‐10 and CPT codes used for cohort analysis.

Category	Condition/Procedure name	Code(s)
Diagnosis	Atherosclerotic heart disease of the native coronary artery	ICD‐10: I25.1
Intervention	Single vessel drug‐eluting stent	ICD‐10‐PCS: 027037(Z,6), 027036(Z,6), 027035(Z,6), 027034(Z,6) CPT: 92928
	Intravascular lithotripsy	HCPCS: C1761 CPT: 92972
	Coronary atherectomy	CPT: 92924, 92933
Primary outcomes	Acute myocardial infarction	ICD‐10: I21
	Stroke	ICD‐10: I63, G45.9
	In‐stent restenosis	ICD‐10‐CM: T82.855
	Revascularization (PCI/CABG)	ICD‐10‐PCS: 0270^a^, 0271^a^, 0272^a^, 0273^a^, 0210^a^, 0211^a^, 0212^a^, 0213^a^ CPT: 3351(0‐4), 3351(6‐9), 3352(1‐3), 3353(3‐6), 9292(0,1), 9292(4,5), 9292(8,9), 9293(7,8), 92941, 9294(3,4)
Secondary outcomes	Major bleeding	ICD‐10: I97.4^a^, I97.5^a^, I97.6^a^, J95.83, R58, T82.83
	Coronary dissection	ICD‐10: I25.4
	Cardiac tamponade	ICD‐10: I31.4
	Hemopericardium	ICD‐10: I31.(2‐4)
	Cardiogenic shock	ICD‐10: I46^a^, R57.0, T81.1^a^
	Stent thrombosis	ICD‐10: T82.867
	Mechanical circulatory support	CPT: 3396(7,8), 3397(0,1,3–8), 3398(0–3), 3399(0–3), 3399(5,7)
	Respiratory failure	ICD‐10: J95.82, J95.89, J96.0
	AKI/CIN	ICD‐10: N14.1, N17, N99.0, T50.8×5

*Note:* Diagnostic, procedural, and outcome definitions used for cohort analyses are shown with corresponding ICD‐10 and CPT codes.

Abbreviations: AKI = acute kidney injury; CABG = coronary artery bypass grafting; CIN = contrast‐induced nephropathy; PCI = percutaneous coronary intervention.

^a^ Indicates inclusion of all available codes beginning with the listed prefix.

Primary outcomes included risk of all‐cause mortality, acute myocardial infarction (MI), stroke (cerebrovascular infarction or transient cerebral ischemic attack), major adverse cardiovascular events (MACE), ISR, and any revascularization via PCI or CABG measured from 1 day until 1 year after index events. Secondary outcomes included risk of periprocedural complications (major bleeding, coronary dissection, cardiac tamponade, hemopericardium, cardiogenic shock, in‐stent thrombosis, mechanical circulatory support, respiratory failure, and contrast‐associated acute kidney injury) measured from 1 day until 30 days following index events. For patients with CAD undergoing single‐vessel DES from 2013 to 2024, we also calculated the annual incidence of IVL and ATH to compare recent trends in their use.

All statistical analyses were performed using the TriNetX Live platform's built‐in analytics, which return de‐identified, aggregate data. Between‐group comparisons were conducted using independent t tests for continuous variables and *χ*² tests for categorical variables. Kaplan–Meier survival analysis was used to compare event‐free survival between cohorts, with differences evaluated by the log‐rank test. Censoring occurred at each patient's last recorded follow‐up encounter, clinical activity, or documented death event within contributing electronic health record data sources. Cox proportional hazards models were used to estimate hazard ratios with 95% confidence intervals, and statistical significance was set at *p* < 0.05. Sensitivity and landmark analyses were then performed for primary outcomes to address potential temporal and selection bias between cohorts. Sensitivity analysis included only IVL and ATH procedures performed during overlapping years (2021−2024) to better compare outcomes of contemporary practices. Overlapping years were chosen based on the first date of IVL coding implementation in our database and allowed for 1 year follow up in both groups. Landmark analysis conditioned on event‐free survival during the first 30 days after index events to assess whether early divergence between our treatment groups persisted until 1 year.

## 3 Results

**Figure 1:**
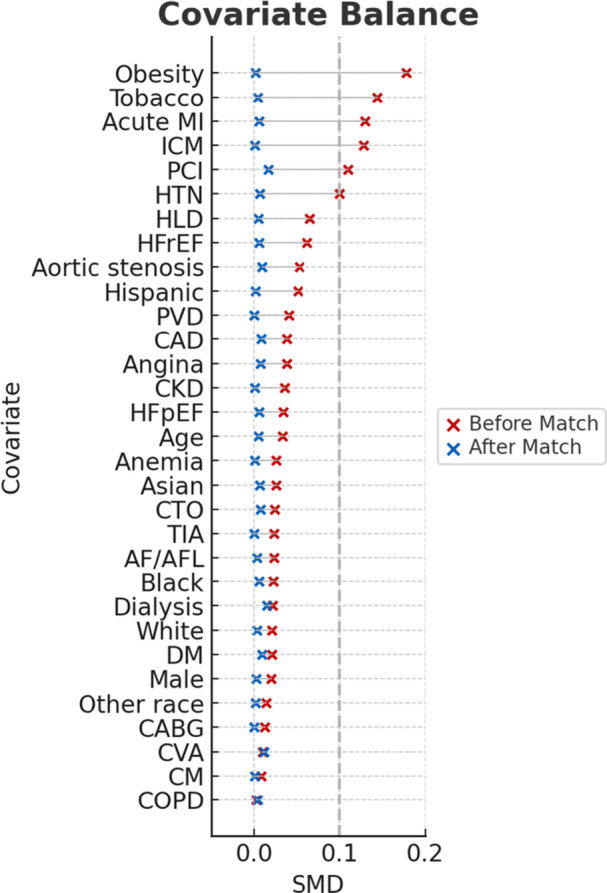
Love Plot for SMD of Baseline Characteristics Before and After Propensity Matching. Covariates are ordered by decreasing order of SMD prior to matching. The vertical dashed line (gray) indicates an absolute SMD = 0.1 threshold for acceptable balance, and all covariates met this threshold after matching (blue). Lesion imaging and procedural details were not available for matching. AF/AFL = atrial fibrillation and flutter, CKD = chronic kidney disease, CABG = coronary artery bypass grafting, CAD = coronary artery disease, CM = cardiomyopathy, COPD = chronic obstructive pulmonary disease, CTO = chronic total occlusion, CVA = ischemic stroke, DM = diabetes mellitus, HFrEF = heart failure with reduced ejection fraction, HFpEF = heart failure with preserved ejection fraction, HLD = hyperlipidemia, HTN = hypertension, ICM = ischemic cardiomyopathy, PCI = percutaneous coronary intervention, PVD = peripheral vascular disease, SMD = standardized mean difference, TIA = transient ischemic attack. [Color figure can be viewed at wileyonlinelibrary.com]

Our registry identified 13,499 patients since 2013 who underwent single‐vessel DES implantation with IVL (*n* = 7026) or ATH (*n* = 6473) before propensity matching. There were 5768 patients in each cohort after matching, and all variables were balanced with no significant differences remaining (*p* > 0.05) (Figure 1, Table 2). Among the ATH cohort, technique‐specific codes were available in 36.1% of cases, of which the majority were rotational (84.2%), followed by directional (13.1%) and orbital (12.4%) ATH. There was a mean follow‐up time of 230 and 285 days, and a median follow‐up time of 280 and 365 days in the IVL and ATH groups, respectively (Table 3).

**Table 2:** Baseline characteristics of patients undergoing DES with IVL versus ATH.

		Before propensity score matching			After propensity score matching		
Characteristics	Codes^a^	IVL	ATH	*p* value	IVL	ATH	*p* value
Number of patients		7026	6473		5768	5768	
Age at Index: mean years ± SD		72.1 ± 9.86	72.4 ± 10.4	0.060	72.2 ± 9.85	72.3 ± 10.3	0.676
Male: *N* (%)		4606 (65.6%)	4186 (64.7%)	0.271	3774 (65.5%)	3767 (65.4%)	0.891
White: *N* (%)		5386 (76.7%)	4908 (75.9%)	0.243	4442 (77.1%)	4414 (76.6%)	0.536
Black or African American: *N* (%)		550 (7.8%)	545 (8.4%)	0.210	465 (8.1%)	469 (8.1%)	0.891
Asian: *N* (%)		366 (5.2%)	302 (4.7%)	0.145	279 (4.8%)	284 (4.9%)	0.829
Hispanic or Latino: *N* (%)		389 (5.5%)	287 (4.4%)	0.003	269 (4.7%)	266 (4.6%)	0.894
Other Race: *N* (%)		165 (2.4%)	139 (2.1%)	0.430	114 (2.0%)	122 (2.1%)	0.599
Hypertension: *N* (%)	I10	5757 (82.0%)	5049 (78.0%)	< 0.001	4558 (79.1%)	4576 (79.4%)	0.679
Angina: *N* (%)	I20	1854 (26.4%)	1601 (24.7%)	0.027	1452 (25.2%)	1437 (24.9%)	0.747
Acute myocardial infarction: *N* (%)	I21	2766 (39.4%)	2961 (45.8%)	< 0.001	2427 (42.1%)	2437 (42.3%)	0.850
Ischemic cardiomyopathy: *N* (%)	I25.5	1191 (17.0%)	1427 (22.1%)	< 0.001	1081 (18.8%)	1074 (18.6%)	0.867
Chronic total occlusion: *N* (%)	I25.82	551 (7.8%)	550 (8.5%)	0.166	464 (8.1%)	462 (8.0%)	0.945
Coronary atherosclerosis: *N* (%)	I25.84	1006 (14.3%)	1015 (15.7%)	0.027	851 (14.8%)	861 (14.9%)	0.793
Aortic stenosis: *N* (%)	I35.0	1161 (16.5%)	1201 (18.6%)	0.002	1013 (17.6%)	996 (17.3%)	0.676
Cardiomyopathy: *N* (%)	I42	1114 (15.9%)	1009 (15.6%)	0.665	872 (15.1%)	875 (15.2%)	0.968
Atrial fibrillation and flutter: *N* (%)	I48	1862 (26.5%)	1781 (27.5%)	0.188	1535 (26.6%)	1535 (26.6%)	1.000
Systolic heart failure: *N* (%)	I50.2	1779 (25.3%)	1817 (28.1%)	< 0.001	1492 (25.9%)	1505 (26.1%)	0.783
Diastolic heart failure: *N* (%)	I50.3	1467 (20.9%)	1265 (19.6%)	0.053	1116 (19.4%)	1134 (19.7%)	0.672
Cerebral infarction: *N* (%)	I63	717 (10.2%)	680 (10.5%)	0.571	582 (10.1%)	597 (10.4%)	0.645
Transient ischemic attack: *N* (%)	G45.9	431 (6.1%)	362 (5.6%)	0.180	322 (5.6%)	319 (5.5%)	0.903
Peripheral vascular disease: *N* (%)	I73.9	1330 (18.9%)	1329 (20.5%)	0.020	1134 (19.7%)	1119 (19.4%)	0.725
Diabetes mellitus: *N* (%)	E08‐E13	3578 (51.0%)	3233 (50.0%)	0.250	2884 (50.1%)	2867 (49.8%)	0.752
Overweight and obesity: *N* (%)	E66	2545 (36.2%)	1809 (28.0%)	< 0.001	1729 (30.0%)	1734 (30.1%)	0.919
Anemia: *N* (%)	D64	2066 (29.4%)	1831 (28.3%)	0.149	1624 (28.2%)	1618 (28.1%)	0.901
Chronic kidney disease: *N* (%)	N18	2390 (34.0%)	2311 (35.7%)	0.041	1985 (34.5%)	1991 (34.6%)	0.906
Other chronic obstructive pulmonary disease: *N* (%)	J44	1429 (20.4%)	1311 (20.3%)	0.896	1124 (19.5%)	1146 (19.9%)	0.606
Hyperlipidemia: *N* (%)	E78.5	5123 (73.0%)	4532 (70.0%)	< 0.001	4058 (70.4%)	4049 (70.3%)	0.854
Tobacco use: *N* (%)	Z72.0	781 (11.1%)	454 (7.0%)	< 0.001	443 (7.7%)	451 (7.8%)	0.781
Bypass: *N* (%)	021^b^	239 (3.4%)	206 (3.2%)	0.474	198 (3.4%)	184 (3.2%)	0.466
Dilation: *N* (%)	027^b^	218 (3.1%)	343 (5.3%)	< 0.001	211 (3.7%)	237 (4.1%)	0.210
Dialysis services: *N* (%)	1012740^b^	347 (4.9%)	351 (5.4%)	0.206	296 (5.1%)	288 (5.0%)	0.734

*Note:* Baseline demographic and clinical characteristics are shown. Categorical data are expressed as number (percentage), and continuous data as mean ± standard deviation. P‐values indicate statistical differences between groups. After propensity score matching, both cohorts were balanced across all variables, as evidenced by nonsignificant p values (*p* > 0.05).

Abbreviations: ATH = atherectomy, DES = drug‐eluting stent, IVL = intravascular lithotripsy.

^a^ Includes ICD‐10, ICD‐10‐PCS, and CPT systems as applicable;

^b^ Includes all subsequent characters for available codes.

**Table 3:** Follow‐up metrics for patients undergoing DES with IVL versus ATH.

Metric	Before matching		After matching	
	IVL	ATH	IVL	ATH
Number of patients	7021	6470	5762	5762
Mean follow‐up (days)	233.0	281.7	230.2	284.7
Standard deviation	140.6	134.7	141.1	132.9
Median follow‐up (days)	288	365	280	365
Interquartile range	274	162	277	148

**Figure 2:**
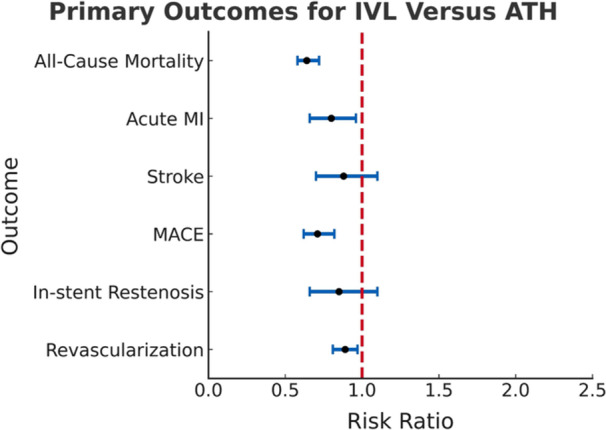
Forest plot for primary outcomes for single‐vessel DES placement using IVL versus ATH at 1 year. RR and 95% CI are shown for each endpoint in blue, with the dashed vertical line representing RR = 1.0 shown in red. RR values < 1.0 favor IVL. IVL was associated with significantly lower all‐cause mortality, acute MI, and MACE compared with ATH. Differences in stroke, in‐stent restenosis, and revascularization (PCI/CABG) were not statistically significant. ATH = atherectomy, CABG = coronary artery bypass grafting, CI = confidence interval, DES = drug‐eluting stent, IVL = intravascular lithotripsy, MACE = major adverse cardiovascular events, MI = myocardial infarction, PCI = percutaneous coronary intervention, RR = risk ratio. [Color figure can be viewed at wileyonlinelibrary.com]

**Figure 3:**
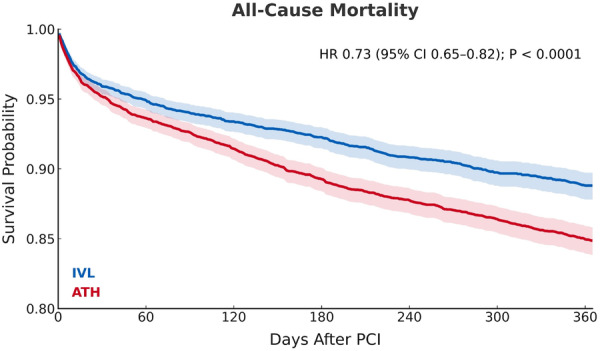
Kaplan‐Meier survival curves for all‐cause mortality at 1 year following single‐vessel PCI with DES placement using either IVL or ATH. Shaded regions indicate 95% confidence intervals. IVL (blue) demonstrated reduced 1‐year mortality compared to ATH (red). Number‐at‐risk tables are not available due to de‐identified aggregate output data. ATH = atherectomy, CI = confidence interval, DES = drug‐eluting stent, HR = hazards ratio, IVL = intravascular lithotripsy, PCI = percutaneous coronary intervention. [Color figure can be viewed at wileyonlinelibrary.com]

**Figure 4:**
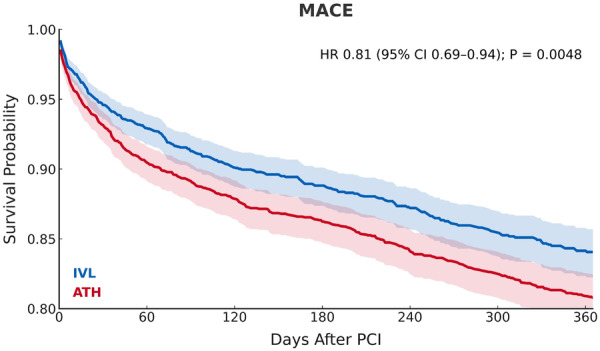
Kaplan‐Meier survival curves for MACE at 1 year following single‐vessel PCI with DES placement using either IVL or ATH. Shaded regions indicate 95% confidence intervals. IVL (blue) demonstrated reduced 1‐year MACE compared to ATH (red). Number‐at‐risk tables are not available due to de‐identified aggregate output data. ATH = atherectomy, CI = confidence interval, DES = drug‐eluting stent, HR = hazards ratio, IVL = intravascular lithotripsy, MACE = major adverse cardiovascular events, PCI = percutaneous coronary intervention. [Color figure can be viewed at wileyonlinelibrary.com]

Significant primary outcomes at 1 year included lower risk of all‐cause mortality (RR 0.64; 95% CI 0.58−0.72; *p* < 0.0001), acute MI (RR 0.80; 95% CI 0.66−0.96; *p* = 0.0166), MACE (RR 0.71; 95% CI 0.62−0.82; *p* < 0.0001) and repeat revascularization (RR 0.89; 95% CI 0.81−0.97; *p* = 0.0107) for DES with IVL compared to ATH (Figure 2, Table 4). There was also a trend toward lower risk of stroke (RR 0.88; 95% CI 0.70−1.1; *p* = 0.2555) and ISR (RR 0.85; 95% CI 0.66−1.10; *p* = 0.2105) in the IVL group, which was not significant. Time‐to‐event analyses further demonstrated significant reductions in all‐cause mortality and MACE with IVL compared to ATH (Figures 3 and 4).

**Table 4:** Primary outcomes for IVL versus ATH with single‐vessel DES placement.

Outcome	Absolute risk		RR	95% CI	*p* value
	IVL	ATH			
All‐cause mortality	496 (8.67%)	770 (13.49%)	0.64	0.58−0.72	< 0.0001
Acute MI	189 (7.01%)	229 (8.79%)	0.80	0.66−0.96	0.0166
Stroke	137 (2.75%)	156 (3.14%)	0.88	0.70−1.10	0.2555
MACE	296 (12.23%)	394 (17.20%)	0.71	0.62−0.82	< 0.0001
In‐stent restenosis	108 (1.87%)	127 (2.20%)	0.85	0.66−1.10	0.2105
Revascularization (PCI/CABG)	720 (12.50%)	813 (14.11%)	0.89	0.81−0.97	0.0107

*Note:* Absolute risk and relative risk with 95% confidence intervals and p‐values for various 1‐year clinical outcomes are shown. IVL was associated with significantly lower all‐cause mortality, acute MI, and MACE compared with ATH. Differences in stroke, in‐stent restenosis, and revascularization were not statistically significant.

Abbreviations: ATH = atherectomy, CABG = coronary artery bypass grafting, CI = confidence interval, DES = drug‐eluting stent, IVL = intravascular lithotripsy, MACE = major adverse cardiovascular events, MI = myocardial infarction, PCI = percutaneous coronary intervention, RR = relative risk.

Sensitivity analysis for primary outcomes at 1 year for IVL and ATH procedures performed during overlapping years redemonstrated significantly lower risk of all‐cause mortality, acute MI, and MACE with IVL compared to ATH (Supporting Information S1: Figure 1‐2, Supporting Information S1: Table 1). Of note, IVL was also significantly associated with reduced ISR in this analysis compared to ATH. Landmark analysis conditioned on event‐free survival at 30 days also redemonstrated significantly lower risk of all‐cause mortality, acute MI, and MACE with IVL compared to ATH, with ISR and repeat revascularization also reaching statistical significance (Supporting Information S1: Figure 3, Supporting Information S1: Table 2). However, the reduction in MACE did not maintain statistical significance with time‐to‐event modeling (Supporting Information S1: Figure 4).

**Figure 5:**
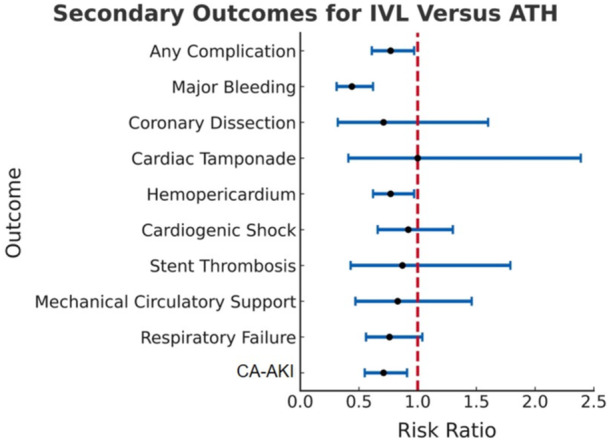
Forest Plot for 30‐Day Procedural Complications Following Single‐Vessel DES Placement Using IVL Versus ATH. RR and 95% CI are shown for each endpoint in blue, with the dashed vertical line representing RR = 1.0 shown in red. RR values < 1.0 favor IVL. IVL was associated with significantly lower rates of any complication, major bleeding, hemopericardium, and CA‐AKI compared with ATH. Differences in coronary dissection, cardiac tamponade, cardiogenic shock, stent thrombosis, mechanical circulatory support, and respiratory failure were not statistically significant. ATH = atherectomy, CA‐AKI = contrast‐associated acute kidney injury, CI = confidence interval, DES = drug‐eluting stent, IVL = intravascular lithotripsy, RR = risk ratio. [Color figure can be viewed at wileyonlinelibrary.com]

There was a significant decrease in risk for any procedural complication at 30 days (RR 0.77; 95% CI 0.61−0.97; *p* = 0.0271), along with significantly less bleeding (RR 0.44; 95% CI 0.31−0.62; *p* < 0.0001), hemopericardium (RR 0.77; 95% CI 0.62−0.97; *p* < 0.0252) and CA‐AKI (RR 0.71; 95% CI 0.55−0.91; *p* = 0.0068) with IVL compared to ATH (Figure 5, Table 5).

**Table 5:** Procedural complications for IVL versus ATH with single‐vessel DES placement.

Outcome	Absolute risk		RR	95% CI	*p* value
	IVL	ATH			
Any complication	124 (4.08%)	143 (5.31%)	0.77	0.61−0.97	0.0271
Major bleeding	44 (0.81%)	99 (1.86%)	0.44	0.31−0.62	< 0.0001
Coronary dissection	≤10 (0.18%)	14 (0.25%)	0.71	0.32−1.60	0.4105
Cardiac tamponade	≤10 (0.18%)	≤10 (0.18%)	1.00	0.41−2.39	0.9909
Hemopericardium	129 (2.24%)	167 (2.90%)	0.77	0.62−0.97	0.0252
Cardiogenic shock	63 (1.22%)	67 (1.32%)	0.92	0.66−1.30	0.6505
Stent thrombosis	14 (0.25%)	16 (0.28%)	0.87	0.43−1.79	0.7096
Mechanical circulatory support	23 (0.45%)	25 (0.54%)	0.83	0.47−1.46	0.5118
Respiratory failure	70 (1.55%)	92 (2.04%)	0.76	0.56−1.04	0.0801
AKI/CIN	99 (2.48%)	139 (3.52%)	0.71	0.55−0.91	0.0068

*Note:* Absolute risk and relative risk with 95% confidence intervals and *p* values for various 30‐day clinical outcomes are shown. IVL was associated with significantly fewer complications overall, including major bleeding, hemopericardium, and AKI/CIN, compared with ATH. Rates of other complications, including coronary dissection, cardiac tamponade, and cardiogenic shock, were low and not significantly different between groups.

Abbreviations: AKI = acute kidney injury, ATH = atherectomy, CI = confidence interval, CIN = contrast‐induced nephropathy, DES = drug‐eluting stent, IVL = intravascular lithotripsy, RR = relative risk.

**Figure 6:**
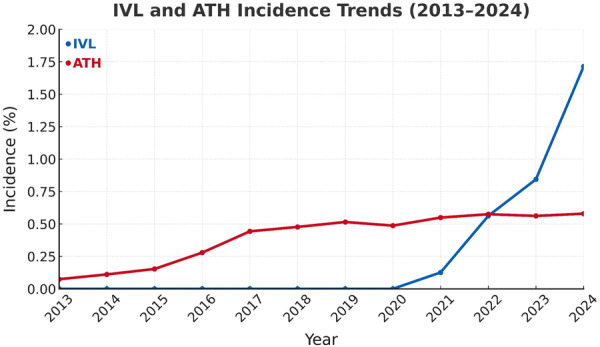
Incidence of IVL and ATH with Single‐Vessel DES Placement (2013–2024). Incidence rates were calculated from all available PCI procedures with single‐vessel DES placement during the time window in the TriNetX Research Network. Utilization of IVL (blue) increased rapidly after 2021 and surpassed ATH (red) after 2022, reflecting accelerated real‐world adoption of IVL for the treatment of coronary lesions. Divergent trends were confirmed on logistic regression analysis (*p* < 0.0001). ATH = atherectomy, DES = drug‐eluting stent, IVL = intravascular lithotripsy, PCI = percutaneous coronary intervention. [Color figure can be viewed at wileyonlinelibrary.com]

Among patients in our registry undergoing single‐vessel DES, the incidence of ATH use increased from 0.11% (238) to 0.58% (1,245) between 2013 and 2024, while the incidence of IVL increased from 0.13% (345) to 1.71% between 2021 and 2024 (Figure 6). The incidence of ATH appears to have plateaued since 2017, while IVL incidence has expanded rapidly and surpassed ATH use after 2022.

## 4 Discussion

In this large‐scale, multicenter registry study of patients undergoing single‐vessel DES implantation, IVL was associated with a significantly lower risk of all‐cause mortality, acute MI, MACE, and repeat revascularization at 1 year compared with ATH. Furthermore, IVL use was associated with fewer periprocedural complications at 30 days, namely due to reductions in bleeding, hemopericardium, and CA‐AKI. These findings support IVL as an increasingly safe and effective alternative to ATH, reflecting active real‐world adoption, although causality cannot be inferred from this study.

Our findings extend the favorable safety profile demonstrated in the DISRUPT CAD I‐IV trials to a larger population and are consistent with recent meta‐analyses demonstrating comparable clinical outcomes between IVL and ATH [4, 5, 6, 7, 11, 12, 13]. The observed decrease in CA‐AKI and bleeding in our IVL cohort is also consistent with potential mechanisms of reduced contrast exposure, vascular access time, and number of device exchanges associated with IVL described in other studies [11, 12, 13]. This has relevance in patients with underlying chronic kidney disease, a population that frequently overlaps with advanced CAD. Our results also corroborate prior observational data reporting low mortality rates with IVL [14, 15, 16]. To our knowledge, however, our study is the first to demonstrate a statistically significant all‐cause mortality benefit associated with IVL compared to ATH.

Additionally, prior studies are limited by sample size and limited follow‐up to 6−12 months. Currently, the largest single registry directly comparing outcomes in IVL or ATH alone includes a total of 2833 patients and reported high rates of procedural success and low complication rates with IVL [17]. We included a total of 11,536 patients in our study after matching, providing even more robust evidence to support similar claims.

To address potential temporal and selection effects, we performed a sensitivity analysis to compare contemporary use of IVL and ATH, and a 30‐day landmark analysis to evaluate whether between‐group separation persisted beyond the acute procedural period. Results remained consistent with our primary analysis, with IVL maintaining significantly lower all‐cause mortality, acute MI, and MACE compared to ATH in both analyses. In the landmark analysis, time‐to‐event demonstrated a nonsignificant trend toward lower MACE with IVL, suggesting that early events within 30 days may have driven the overall hazard difference (Supporting Information S1: Figure 4). In contrast, reductions in ISR and repeat revascularization associated with IVL reached statistical significance, consistent with the expected timing of mid‐term stent failure events (Supporting Information S1: Table 2). A plausible explanation for this finding is the higher 30‐day mortality observed with ATH, which may have obscured later nonfatal differences, as deaths are treated as censoring events in Kaplan–Meier analyses (Figure 3). Altogether, the consistency of these results across both subgroup analyses supports our main findings and suggests that the observed primary outcomes with IVL are not solely driven by differences in contemporary practice patterns or selection bias.

### 4.1 Study Limitations

This study has several limitations inherent to its observational design using a large‐scale registry. First, while propensity score matching was used to balance known confounding variables, there may be unmeasured confounders that could influence outcomes. These include important lesion‐specific and procedural factors such as calcium morphology, burr or balloon sizing, intravascular imaging use, access or anticoagulation strategies, and operator experience, all of which are known to impact clinical outcomes but were unavailable in our study [2, 3, 9, 17]. The lack of these data precludes deeper mechanistic insight into how IVL may reduce mortality or procedural complications.

Second, our analysis lacked detailed procedural data and lesion‐specific characteristics that typically guide the selection of IVL versus ATH. For instance, ATH is generally preferred for modifying device‐uncrossable, long, diffuse, or eccentric calcified lesions, whereas IVL is often preferred for balloon‐crossable, focal, short, or circumferential lesions [18]. While there are overlapping indications, the device used is often based on these subtle differences, which we were unable to account for. Similarly, the choice among different ATH techniques (e.g., rotational, orbital, directional, or laser) depends on lesion morphology and technical considerations that were not captured in this dataset, introducing potential selection bias. Technique‐specific ATH codes were inconsistently reported across institutions and present in only a minority of cases, limiting our ability to accurately characterize modality use. Hybrid ATH‐IVL cases were also excluded from our study, which may underrepresent complex coronary lesions where sequential or bailout ATH and IVL are sometimes required. Furthermore, our study included all patients with CAD undergoing single‐vessel PCI with DES, even though IVL and ATH are predominantly used for heavily calcified lesions. We decided to include these patients to maximize our sample size, although this may limit the generalizability to populations with severe calcifications. Since IVL may have been the chosen strategy for patients with favorable, albeit unmeasured, plaque characteristics, the possibility of residual treatment‐selection bias should be considered when interpreting these findings.

Third, since TriNetX aggregates data at the site level and does not provide patient‐level times to events or censoring records, we were unable to display standard “number at risk” tables beneath Kaplan–Meier curves. The platform does not distinguish whether censoring reflects administrative truncation, data refresh intervals, loss to follow‐up, or death, which may contribute to variable follow‐up duration and limit the generalizability of our findings. These constraints also apply to the sensitivity and landmark analyses, which should be interpreted as hypothesis‐generating and supportive of our main findings rather than confirmatory.

Finally, our analysis relied on coding data provided by participating health care organizations, which may be subject to errors or misclassification that led to less precise primary or secondary outcomes. For example, although we were able to describe the risks of undergoing any subsequent revascularization by 1 year, we were unable to specify target‐vessel revascularization based on the available set of codes.

Despite these limitations, our analysis does include the largest reported population study comparing IVL and ATH outcomes, representing a strong potential for IVL favorability, when deemed feasible. These novel strengths ultimately highlight the need for prospective, randomized controlled trials to address temporal confounding and other unmeasured variables that may influence our observed differences in outcomes. Such trials will be essential to validate our findings and assess for causality between IVL and improved outcomes for severely calcified coronary lesions.

## 5 Conclusions

In this large, propensity‐matched registry study, IVL was associated with a significant reduction in all‐cause mortality, acute MI, MACE, and revascularization at 1 year compared to ATH. IVL was also linked to fewer periprocedural complications, including less bleeding and CA‐AKI. These findings highlight a strong association between IVL use and improved clinical outcomes, suggesting IVL is a safe and effective alternative to ATH for plaque modification in patients with CAD, supported by rapid real‐world adoption. This study provides robust, large‐scale evidence of IVL benefits and supports IVL as a preferred strategy in contemporary practice, underscoring the need for randomized, image‐guided, and anatomically stratified trials to validate these findings. Central Illutration 1

**Central Illutration 1.**
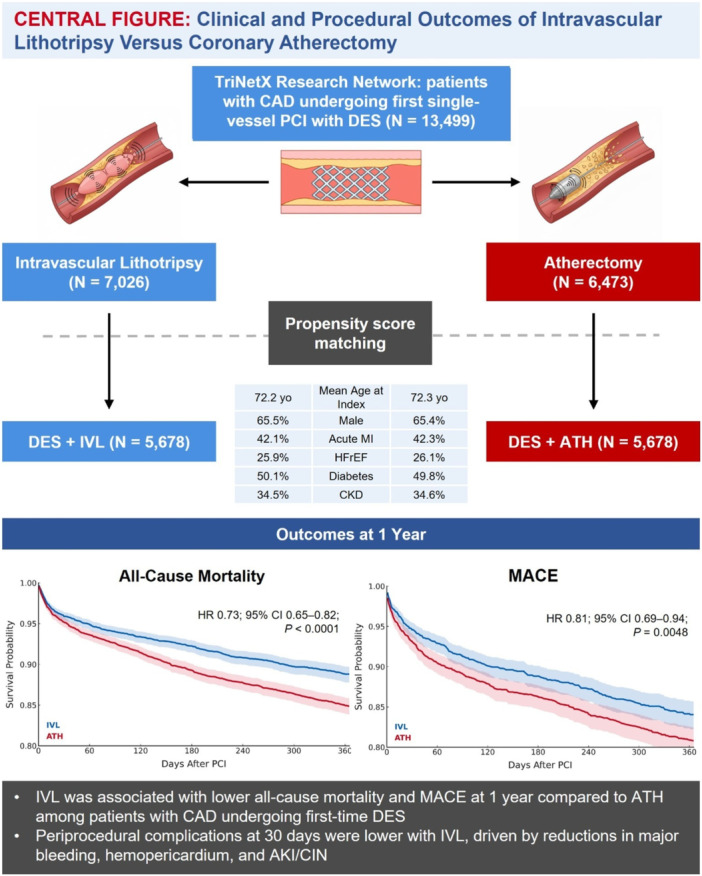
Clinical and procedural outcomes of IVL versus coronary atherectomy. Among patients with CAD undergoing first‐time DES placement, IVL was associated with lower all‐cause mortality and MACE at 1 year, along with decreased periprocedural complications of major bleeding, hemopericardium, and CA‐AKI at 30 days, compared to ATH. ATH = atherectomy, CA‐AKI = contrast‐associated acute kidney injury, CAD = coronary artery disease, CI = confidence interval, CKD = chronic kidney disease, DES = drug‐eluting stent, HFrEF = heart failure with reduced ejection fraction, HR = hazard ratio, IVL = intravascular lithotripsy, MACE = major adverse cardiovascular events, MI = myocardial infarction, PCI = percutaneous coronary intervention. [Color figure can be viewed at wileyonlinelibrary.com]

## Artificial Intelligence Generated Content

No AI tools were used for the study design, data analysis, or performance of the research.

## Disclosure

The authors have nothing to report.

## Ethics Statement

The University of Iowa Health Care IRB Chair or Chair Designee has determined that this project does not meet the regulatory definition of human subject's research and does not require review by the IRB, because this activity is based on evaluation of de‐identified outcome data using TriNetX.

## Conflicts of Interest

The authors declare no conflicts of interest.

## Supporting information


**Table S1.** Sensitivity Analysis for Primary Outcomes of IVL Versus ATH with Single‐Vessel DES Placement from 2021‐2024. **Table S2.** Landmark Analysis for Primary Outcomes of IVL Versus ATH with Single‐Vessel DES Placement. **Figure S1.** Kaplan‐Meier survival curves for all‐cause mortality at 1 year following single‐vessel PCI with DES placement using either IVL or ATH from 2021‐2024. **Figure S2.** Kaplan‐Meier survival curves for MACE at 1 year following single‐vessel PCI with DES placement using either IVL or ATH from 2021‐2024. **Figure S3.** Kaplan‐Meier survival curves for all‐cause mortality from 30 days to 1 year following single‐vessel PCI with DES placement using either IVL or ATH. Shaded regions indicate 95% confidence intervals. **Figure S4.** Kaplan‐Meier survival curves for MACE from 30 days to 1 year following single‐vessel PCI with DES placement using either IVL or ATH. Shaded regions indicate 95% confidence intervals.
