# Supplementary material for: Intravascular Lithotripsy Is Associated With Superior Clinical Outcomes Compared to Atherectomy: A Large‐Scale, Propensity‐Matched Analysis
Source: Catheter Cardiovasc Interv. 2026 Mar 16;107(6):1934–44. doi: 10.1002/ccd.70464 (PMC13176710; doi:10.1002/ccd.70464)
Supplement: Supplementary file 1 — Table S1. Sensitivity Analysis for Primary Outcomes of IVL Versus ATH with Single‐Vessel DES Placement from 2021‐2024. Table S2. Landmark Analysis for Primary Outcomes of IVL Versus ATH with Single‐Vessel DES Placement. Figure S1. Kaplan‐Meier survival curves for all‐cause mortality at 1 year following single‐vessel PCI with DES placement using either IVL or ATH from 2021‐2024. Figure S2. Kaplan‐Meier survival curves for MACE at 1 year following single‐vessel PCI with DES placement using either IVL or ATH from 2021‐2024. Figure S3. Kaplan‐Meier survival curves for all‐cause mortality from 30 days to 1 year following single‐vessel PCI with DES placement using either IVL or ATH. Shaded regions indicate 95% confidence intervals. Figure S4. Kaplan‐Meier survival curves for MACE from 30 days to 1 year following single‐vessel PCI with DES placement using either IVL or ATH. Shaded regions indicate 95% confidence intervals. [file CCD-107-1934-s001.docx]

**Supplemental Tables**

**Table S1.** Sensitivity Analysis for Primary Outcomes of IVL Versus ATH with Single-Vessel DES Placement from 2021-2024

| **Outcome** | **Absolute Risk** | | **RR** | **95% CI** | **p-value** |
| --- | --- | --- | --- | --- | --- |
|  | **IVL** | **ATH** |  |  |  |
| All-Cause Mortality | 326 (10.52%) | 407 (13.15%) | 0.80 | 0.70 – 0.92 | 0.0013 |
| Acute MI | 75 (6.02%) | 120 (10.22%) | 0.59 | 0.45 – 0.78 | 0.0001 |
| Stroke | 74 (2.79%) | 89 (3.37%) | 0.83 | 0.61 – 1.12 | 0.2223 |
| MACE | 128 (11.48%) | 176 (17.20%) | 0.67 | 0.54 – 0.82 | 0.0002 |
| In-stent Restenosis | 81 (2.60%) | 117 (3.75%) | 0.69 | 0.52 – 0.92 | 0.0093 |
| Revascularization (PCI/CABG) | 402 (12.89%) | 424 (13.60%) | 0.95 | 0.84 – 1.08 | 0.4112 |

Absolute risk and relative risk with 95% confidence intervals and p-values for various one-year clinical outcomes for IVL and ATH from 2021-2024 are shown. IVL was associated with significantly lower all-cause mortality, acute MI, MACE, and ISR compared with ATH. Differences in stroke and repeat revascularization were not statistically significant. *ATH = atherectomy; CABG = coronary artery bypass grafting; CI = confidence interval; DES = drug-eluting stent; IVL = intravascular lithotripsy; MACE = major adverse cardiovascular events; MI = myocardial infarction; PCI = percutaneous coronary intervention; RR = relative risk.*

**Table S2.** Landmark Analysis for Primary Outcomes of IVL Versus ATH with Single-Vessel DES Placement

| **Outcome** | **Absolute Risk** | | **RR** | **95% CI** | **p-value** |
| --- | --- | --- | --- | --- | --- |
|  | **IVL** | **ATH** |  |  |  |
| All-Cause Mortality | 364 (5.69%) | 564 (8.94%) | 0.64 | 0.56–0.72 | < 0.0001 |
| Acute MI | 119 (4.01%) | 147 (5.10%) | 0.79 | 0.62–1.00 | 0.0452 |
| Stroke | 117 (2.05%) | 140 (2.46%) | 0.83 | 0.65–1.06 | 0.1428 |
| MACE | 207 (7.92%) | 278 (11.19%) | 0.71 | 0.60–0.84 | < 0.0001 |
| In-stent Restenosis | 128 (1.93%) | 194 (2.92%) | 0.66 | 0.53–0.82 | 0.0002 |
| Revascularization (PCI/CABG) | 407 (6.11%) | 545 (8.18%) | 0.75 | 0.66–0.85 | < 0.0001 |

Absolute risk and relative risk with 95% confidence intervals and p-values for various one-year clinical outcomes with measurements starting 30 days after the index event are shown. IVL was associated with significantly lower all-cause mortality, acute MI, MACE, ISR, and repeat revascularization compared with ATH. Differences in stroke were not statistically significant.
*ATH = atherectomy; CABG = coronary artery bypass grafting; CI = confidence interval; DES = drug-eluting stent; IVL = intravascular lithotripsy; MACE = major adverse cardiovascular events; MI = myocardial infarction; PCI = percutaneous coronary intervention; RR = relative risk.*

**Supplemental Figures
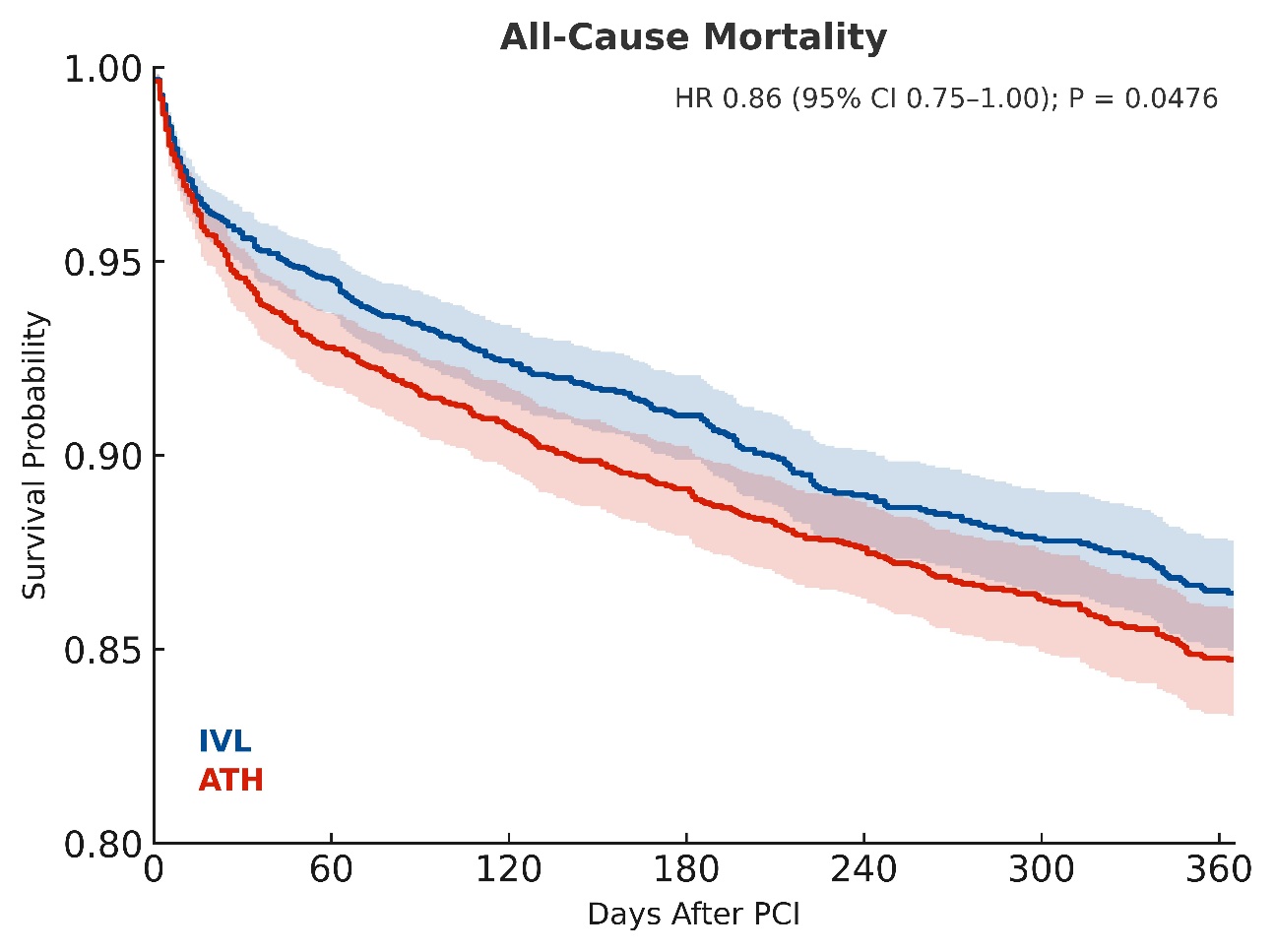
**

**Figure S1.** Kaplan-Meier survival curves for all-cause mortality at 1 year following single-vessel PCI with DES placement using either IVL or ATH from 2021-2024. Shaded regions indicate 95% confidence intervals. IVL (blue) demonstrated reduced 1-year mortality compared to ATH (red). Number-at-risk tables are not available due to de-identified aggregate output data. *ATH = atherectomy; CI = confidence interval; DES = drug-eluting stent; HR = hazards ratio; IVL = intravascular lithotripsy; PCI = percutaneous coronary intervention.*

**
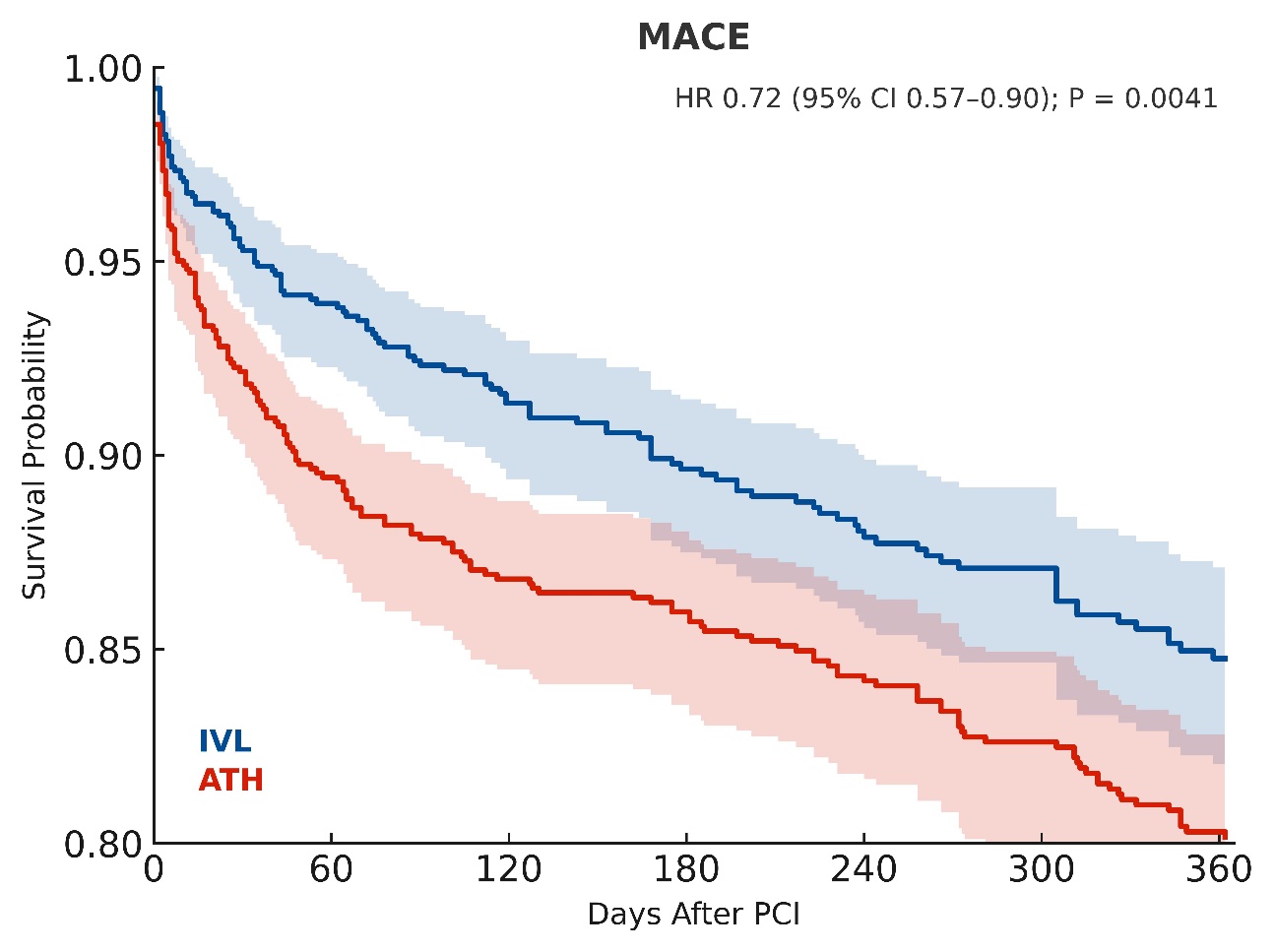
**

**Figure S2.** Kaplan-Meier survival curves for MACE at 1 year following single-vessel PCI with DES placement using either IVL or ATH from 2021-2024. Shaded regions indicate 95% confidence intervals. IVL (blue) demonstrated reduced 1-year MACE compared to ATH (red). Number-at-risk tables are not available due to de-identified aggregate output data. *ATH = atherectomy; CI = confidence interval; DES = drug-eluting stent; HR = hazards ratio; IVL = intravascular lithotripsy; MACE = major adverse cardiovascular events; PCI = percutaneous coronary intervention.*

**
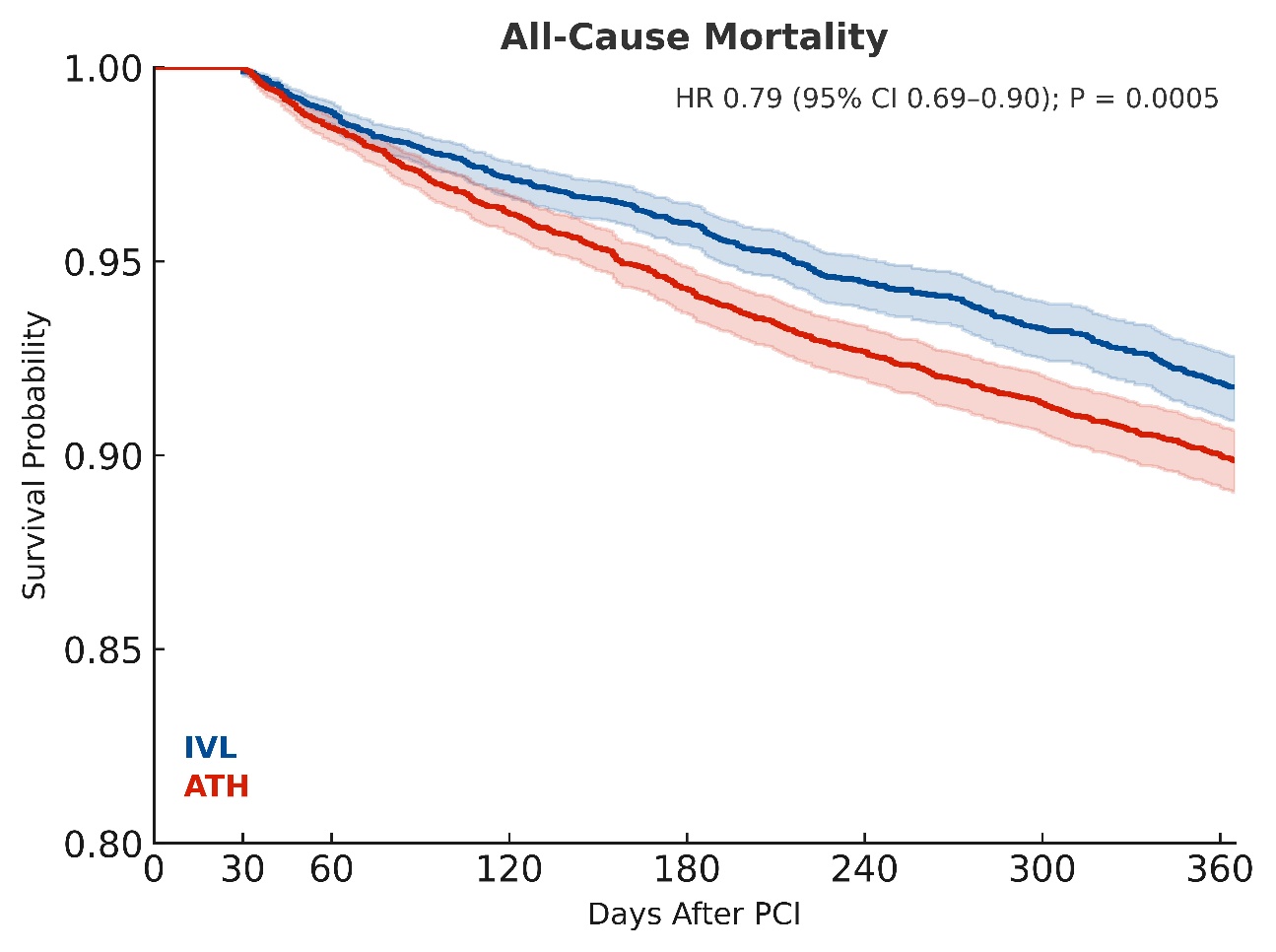

Figure S3.** Kaplan-Meier survival curves for all-cause mortality from 30 days to 1 year following single-vessel PCI with DES placement using either IVL or ATH. Shaded regions indicate 95% confidence intervals. IVL (blue) demonstrated reduced 1-year mortality compared to ATH (red). Number-at-risk tables are not available due to de-identified aggregate output data. *ATH = atherectomy; CI = confidence interval; DES = drug-eluting stent; HR = hazards ratio; IVL = intravascular lithotripsy; PCI = percutaneous coronary intervention.*

**
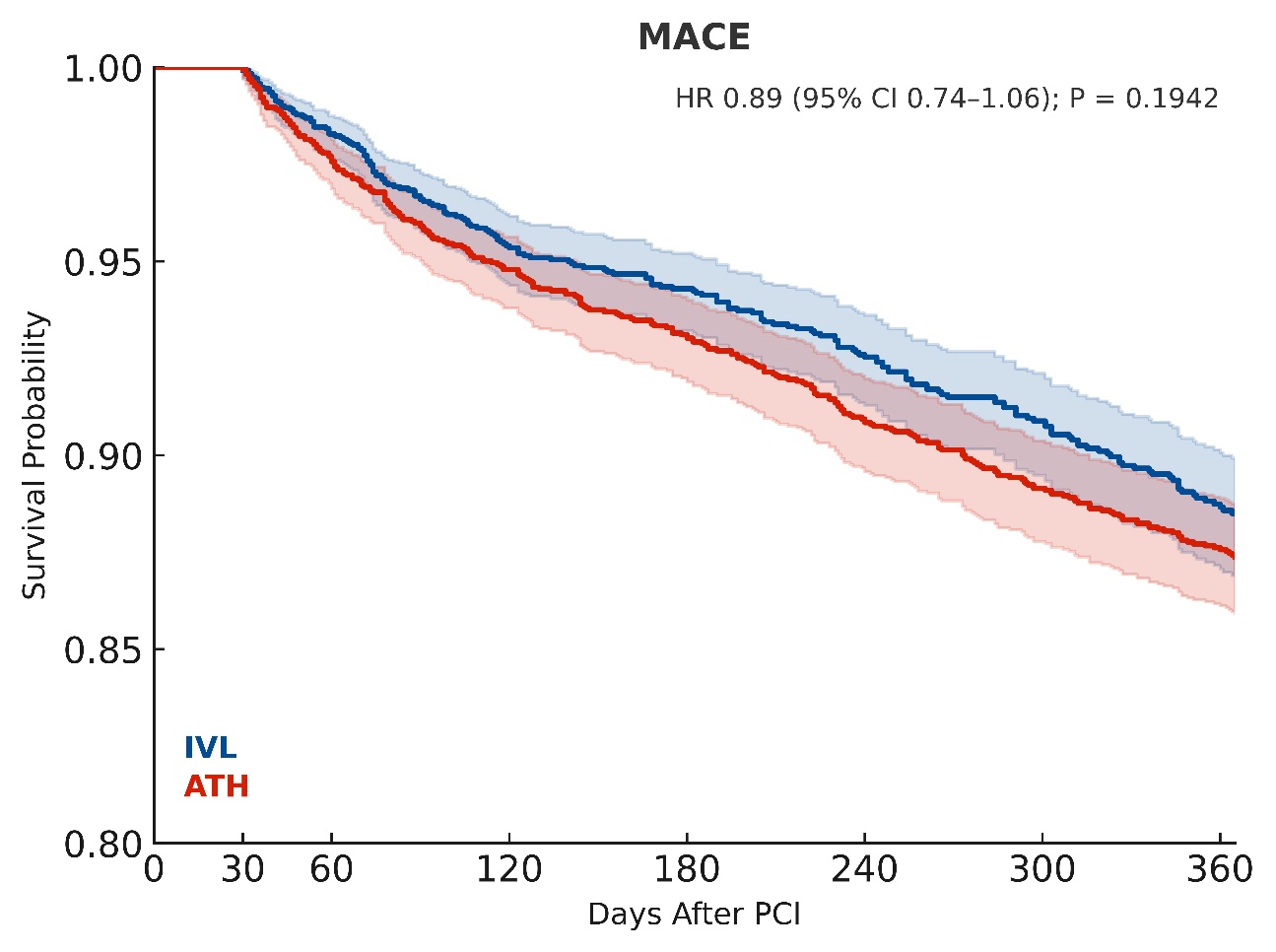
**

**Figure S4.** Kaplan-Meier survival curves for MACE from 30 days to 1 year following single-vessel PCI with DES placement using either IVL or ATH. Shaded regions indicate 95% confidence intervals. IVL (blue) demonstrated a trend towards reduced 1-year MACE compared to ATH (red). Number-at-risk tables are not available due to de-identified aggregate output data. *ATH = atherectomy; CI = confidence interval; DES = drug-eluting stent; HR = hazards ratio; IVL = intravascular lithotripsy; MACE = major adverse cardiovascular events; PCI = percutaneous coronary intervention.*
